# Visualizing Association of the Retroviral Gag Protein with Unspliced Viral RNA in the Nucleus

**DOI:** 10.1128/mBio.00524-20

**Published:** 2020-04-07

**Authors:** Rebecca J. Kaddis Maldonado, Breanna Rice, Eunice C. Chen, Kevin M. Tuffy, Estelle F. Chiari, Kelly M. Fahrbach, Thomas J. Hope, Leslie J. Parent

**Affiliations:** a Department of Medicine, Penn State College of Medicine, Hershey, Pennsylvania, USA; b Department of Microbiology and Immunology, Penn State College of Medicine, Hershey, Pennsylvania, USA; c Department of Cell and Molecular Biology, Northwestern University, Chicago, Illinois, USA; Columbia University/HHMI

**Keywords:** Gag proteins, RNA trafficking, Rous sarcoma virus, genomic RNA packaging, live cell imaging, nucleocytoplasmic trafficking, retrovirus assembly

## Abstract

Packaging of genomic RNA (gRNA) by retroviruses is essential for infectivity, yet the subcellular site of the initial interaction between the Gag polyprotein and gRNA remains poorly defined. Because retroviral particles are released from the plasma membrane, it was previously thought that Gag proteins initially bound to gRNA in the cytoplasm or at the plasma membrane. However, the Gag protein of the avian retrovirus Rous sarcoma virus (RSV) undergoes active nuclear trafficking, which is required for efficient gRNA encapsidation (L. Z. Scheifele, R. A. Garbitt, J. D. Rhoads, and L. J. Parent, Proc Natl Acad Sci U S A 99:3944–3949, 2002, https://doi.org/10.1073/pnas.062652199; R. Garbitt-Hirst, S. P. Kenney, and L. J. Parent, J Virol 83:6790–6797, 2009, https://doi.org/10.1128/JVI.00101-09). These results raise the intriguing possibility that the primary contact between Gag and gRNA might occur in the nucleus. To examine this possibility, we created a RSV proviral construct that includes 24 tandem repeats of MS2 RNA stem-loops, making it possible to track RSV viral RNA (vRNA) in live cells in which a fluorophore-conjugated MS2 coat protein is coexpressed. Using confocal microscopy, we observed that both wild-type Gag and a nuclear export mutant (Gag.L219A) colocalized with vRNA in the nucleus. In live-cell time-lapse images, the wild-type Gag protein trafficked together with vRNA as a single ribonucleoprotein (RNP) complex in the nucleoplasm near the nuclear periphery, appearing to traverse the nuclear envelope into the cytoplasm. Furthermore, biophysical imaging methods suggest that Gag and the unspliced vRNA physically interact in the nucleus. Taken together, these data suggest that RSV Gag binds unspliced vRNA to export it from the nucleus, possibly for packaging into virions as the viral genome.

## Abstract (precis)

Retroviruses cause severe diseases in animals and humans, including cancer and acquired immunodeficiency syndromes. To propagate infection, retroviruses assemble new virus particles that contain viral proteins and unspliced vRNA to use as gRNA. Despite the critical requirement for gRNA packaging, the molecular mechanisms governing the identification and selection of gRNA by the Gag protein remain poorly understood. In this report, we demonstrate that the Rous sarcoma virus (RSV) Gag protein colocalizes with unspliced vRNA in the nucleus in the interchromatin space. Using live-cell confocal imaging, RSV Gag and unspliced vRNA were observed to move together from inside the nucleus across the nuclear envelope, suggesting that the Gag-gRNA complex initially forms in the nucleus and undergoes nuclear export into the cytoplasm as a viral ribonucleoprotein (vRNP) complex.

## INTRODUCTION

The retroviral Gag polyprotein orchestrates the production of virus particles, which are released from the plasma membrane of infected cells (1, 2, and reviewed in reference 3). After virus particle maturation, the Gag polyprotein is cleaved into the matrix (MA), capsid (CA), and nucleocapsid (NC) proteins, as well as smaller cleavage products that vary among viruses (reviewed in reference 4). Infectious virus particles contain two copies of the genomic vRNA (gRNA), linked by a noncovalent dimer near the 5′ end of the genome (reviewed in reference 5). During virus entry, gRNA is converted to double-stranded DNA, which integrates into areas of open cellular chromatin to form the provirus (reviewed in references 6 and 7). Host-encoded RNA polymerase II (Pol II) synthesizes full-length vRNA, which is cotranscriptionally spliced into subgenomic mRNAs or remains full length and is exported from the nucleus for use as mRNA for the translation of Gag and Gag-Pol or as gRNA for encapsidation into nascent virus particles (reviewed in reference 8).

A longstanding question in the field is the mechanism by which Gag locates and selectively binds unspliced viral gRNA for packaging. This process is especially challenging given that vRNA makes up less than 1% of the total RNA in the infected cell (9). Historically, it was thought that recognition and binding of gRNA by Gag occurred in the cytoplasm or at the plasma membrane. However, given that retroviral RNA synthesis occurs in the nucleus and that the Gag proteins of many retroviruses, including Rous sarcoma virus (RSV) (1, 2, 10–15), feline immunodeficiency virus (16), foamy virus (17–23), human immunodeficiency virus type 1 (HIV-1) (13), Mason-Pfizer monkey virus (24–26), mouse mammary tumor virus (13, 27), and murine leukemia virus (28), are present in the nucleus (29), it is plausible to hypothesize that retroviral Gag proteins associate with gRNA in the nucleus.

The mechanisms governing Gag nuclear localization have been studied most extensively for RSV (1, 2, 10–14, 29–31). Nuclear localization signals (NLSs) reside in the MA and NC domains, and a Crm1-dependent nuclear export signal (NES) was identified in the p10 domain upstream of CA (1, 10–12, 14, 31). To examine the function of RSV Gag nuclear trafficking, genetic loss- and gain-of-function experiments were performed. Mutants with reduced nuclear trafficking produced virus particles deficient in gRNA incorporation and restoration of nuclear trafficking using a heterologous NLS-rescued gRNA packaging to nearly wild-type levels (1, 2, 14, 31), suggesting that nuclear trafficking of RSV Gag is required for gRNA incorporation. *In vitro* assays revealed that RNA facilitates the binding of RSV Gag to the nuclear export complex Crm1-RanGTP, suggesting that the Gag-nucleic acid association induces a conformational change exposing the NES in p10 (11). These data form the basis of a model in which RSV Gag enters the nucleus, locates and binds unspliced vRNA, undergoes a structural change allowing binding of the p10 NES to the Crm1-RanGTP export complex, and transports vRNA into the cytoplasm and, subsequently, to the plasma membrane (11).

In the current studies, we directly examined whether RSV Gag associates with vRNA in the nucleus using live-cell confocal microscopy to image vRNP complexes. These data demonstrate that Gag and vRNA colocalized in the nucleus and moved together as a single vRNP across the nuclear membrane toward the cytoplasm. These observations were supported by studies using single-molecule fluorescent *in situ* hybridization (smFISH) to specifically detect unspliced vRNA, which colocalized in three dimensions with wild-type Gag in the 4′,6-diamidino-2-phenylindole (DAPI)-poor interchromatin space. Restricting RSV Gag to the nucleus by mutating the p10 NES resulted in a high degree of Gag-vRNA nuclear colocalization and selective binding to vRNA. These data provide evidence in support of a novel mechanism by which retroviruses select their unspliced RNA genomes in the nucleus.

## RESULTS

**FIG 1:**
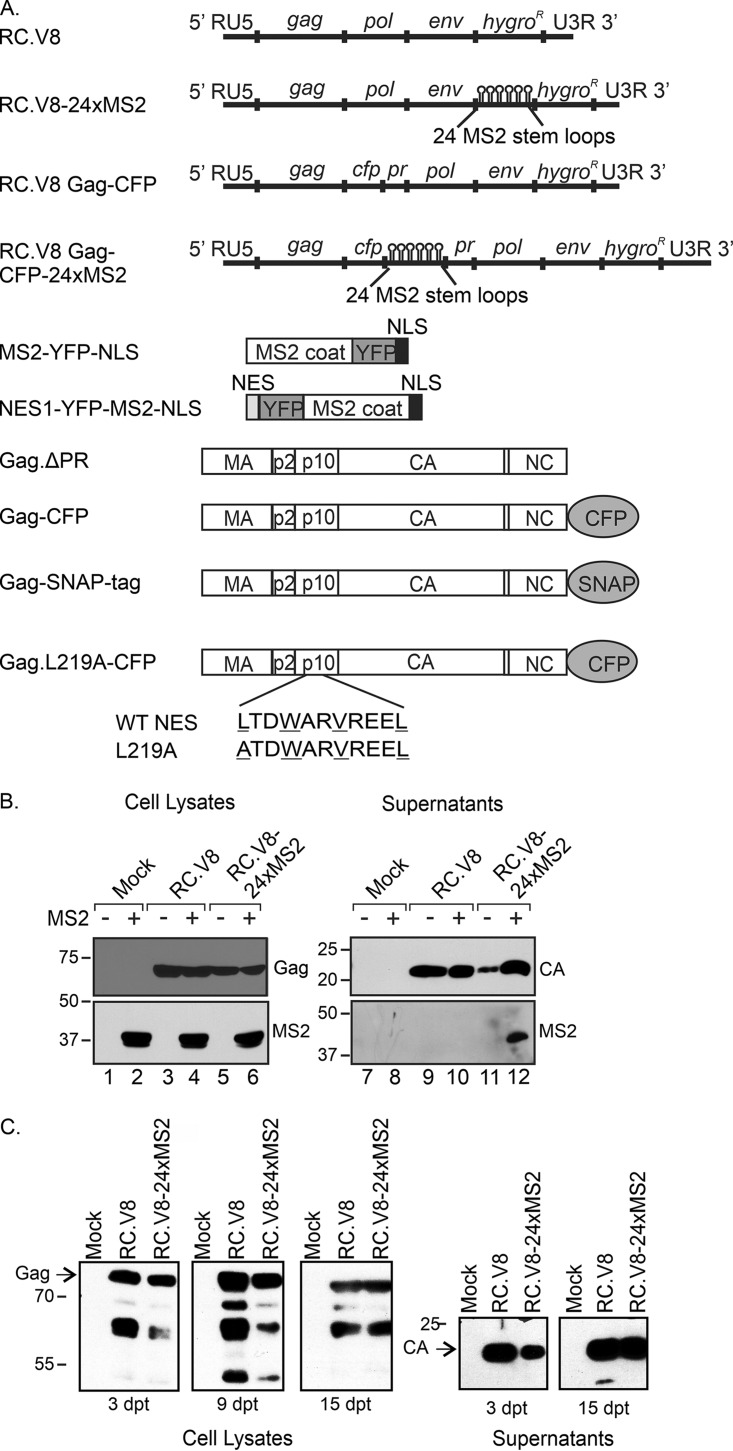
Schematic diagram and characterization of MS2-containing proviral constructs. (A) RNAs and proteins used in this paper. The pRC.V8, pRC.V8-24xMS2, and pRC.V8 Gag-CFP expression plasmids are derived from pRCAN, which contains the hygromycin gene (*hygro*) in place of the *src* coding sequence. Twenty-four tandem repeats of the 19 nucleotide MS2 phage RNA stem-loops (36) were inserted into pRC.V8 upstream of the hygromycin coding region to create pRC.V8-24xMS2, causing the loops to be present in both spliced and unspliced vRNA. To create pRC.V8 Gag-CFP, CFP was fused to the C terminus NC, and CFP contains stop codons to prevent the translation of a Gag-CFP-pr-pol protein. pRC.V8 Gag-CFP-24xMS2 was created by inserting 24 copies of MS2 loops into the BstBI and SpeI sites in pRC.V8 Gag-CFP, and the loops are only present in the unspliced vRNA. The pMS2-YFP-NLS expression plasmid, under the control of the polymerase II (Pol II) promoter, contains an NLS that targets MS2-YFP-NLS coat protein to the nucleus, where it binds RNA cotranscriptionally (34). pNES1-YFP-MS2-NLS encodes the Rev NES and well as an NLS to help reduce background fluorescence in the nucleus (49). Wild-type Gag-CFP, wild-type Gag.ΔPR, and Gag.L219A-CFP were previously described (12, 31). Gag.L219A-CFP contains a point mutation in the coding sequence of the p10 domain nuclear export signal, causing the mutant protein to accumulate in the nucleus and within nuclear foci and nucleoli (12, 14). (B) Whole-cell lysates (lanes 1 to 6) and viral particles (lanes 7 to 12) were collected 48 h posttransfection (pt). Western blotting against RSV (rabbit polyclonal antibody) (top row) and MS2 coat protein (3H4 monoclonal antibody; bottom row) was performed to assess the viral protein production and MS2-YFP-NLS incorporation in viral particles. (C) Whole-cell lysates were collected every 3 days for 12 days. Western blotting for RSV Gag was performed at the end of the collection period. On both days 3 and 12 pt, RC.V8 and RC.V8-24xMS2 (RC.V8-24xMS2) cells produced Gag protein. The contrast and brightness were adjusted across the entire image to remove background from the film.

Genetic and biochemical evidence suggests that RSV Gag nuclear trafficking is necessary for efficient packaging of gRNA (2, 11), leading to the hypothesis that Gag binds the vRNA genome in the nucleus. To test this hypothesis, we adopted an imaging approach to visualize the subcellular location of Gag-vRNA interactions in living cells. To this end, 24 tandem repeats encoding MS2 bacteriophage RNA stem-loops were inserted near the 3′ end of the RSV genome in the pRC.V8 proviral construct (32, 33) to create RC.V8-24xMS2 (Fig. 1A). Binding of up to 48 fluorophore-tagged MS2 coat proteins to the vRNA allows RNA detection at single-molecule resolution using confocal microscopy (34–36). A similar approach has been used to study RNA trafficking of other retroviruses, including HIV-1 (37–42), feline immunodeficiency virus (FIV) (43), and murine leukemia virus (MLV) (44), and the retrotransposons Ty1 (45), Ty3 (46), and Tf1 (47).

### Genomic RNA packaging and infectivity of the RC.V8-24xMS2 virus.

To determine whether inserting MS2 RNA stem-loops altered viral gene expression or interfered with the incorporation of gRNA into virus particles, we expressed wild-type RC.V8 (lacking the MS2 cassette) or pRC.V8-24xMS2 (containing the MS2 sequence in the 3′ exon) in QT6 cells (48). Following transfection of pRC.V8 or pRC.V8-24xMS2, the expression of Gag proteins was detected in cell lysates, and virus particles were released from the cells, as indicated by the presence of CA in the supernatants (Fig. 1B, lanes 9 to 12), although the amount of Gag expression and virus released from pRC.V8-24xMS2 appeared to be slightly decreased compared to that of pRC.V8 (Fig. 1B). Cotransfection of cells with MS2-yellow fluorescent protein-NLS (MS2-YFP-NLS) and either pRC.V8 or pRC.V8-24xMS2 resulted in the expression of MS2 in the appropriate cell lysates (Fig. 1B, lanes 2, 4, and 6). The MS2-YFP-NLS protein was incorporated into virus particles released from cells coexpressing pMS2-YFP-NLS and pRC.V8-24xMS2 but not in cells transfected with pRC.V8, as expected (Fig. 1B, lanes 10 and 12). These results indicate that the MS2-YFP-NLS coat protein specifically binds RC.V8-24xMS2 gRNA in cells and is packaged into virus particles, recapitulating the normal gRNA packaging pathway.

Next, an infectivity assay was performed to assess whether cells transfected with pRC.V8-24xMS2 were capable of undergoing integration and producing a spreading infection, resulting in persistent Gag protein expression. At 3, 9, and 12 days posttransfection (dpt), RSV Gag was detected in cell lysates expressing RC.V8 or RC.V8-24xMS2, and virus particles were detected in culture supernatants at 3 and 15 dpt (Fig. 1C), indicating that productive infection was established. There appeared to be a mild delay in infectivity of the MS2-containing viral construct, as indicated by the slightly reduced levels of Gag in the cell lysates and CA in the medium at 3 and 9 dpt for RC.V8-24xMS2 compared to the levels of RC.V8. Although the levels of Gag expression were the same by 15 dpt, it is possible that a portion of the MS2 cassette was deleted spontaneously in a subpopulation of the infected cells.

### Visualization of MS2 stem-loop-containing RNAs bound to MS2-YFP-NLS.

**FIG 2:**
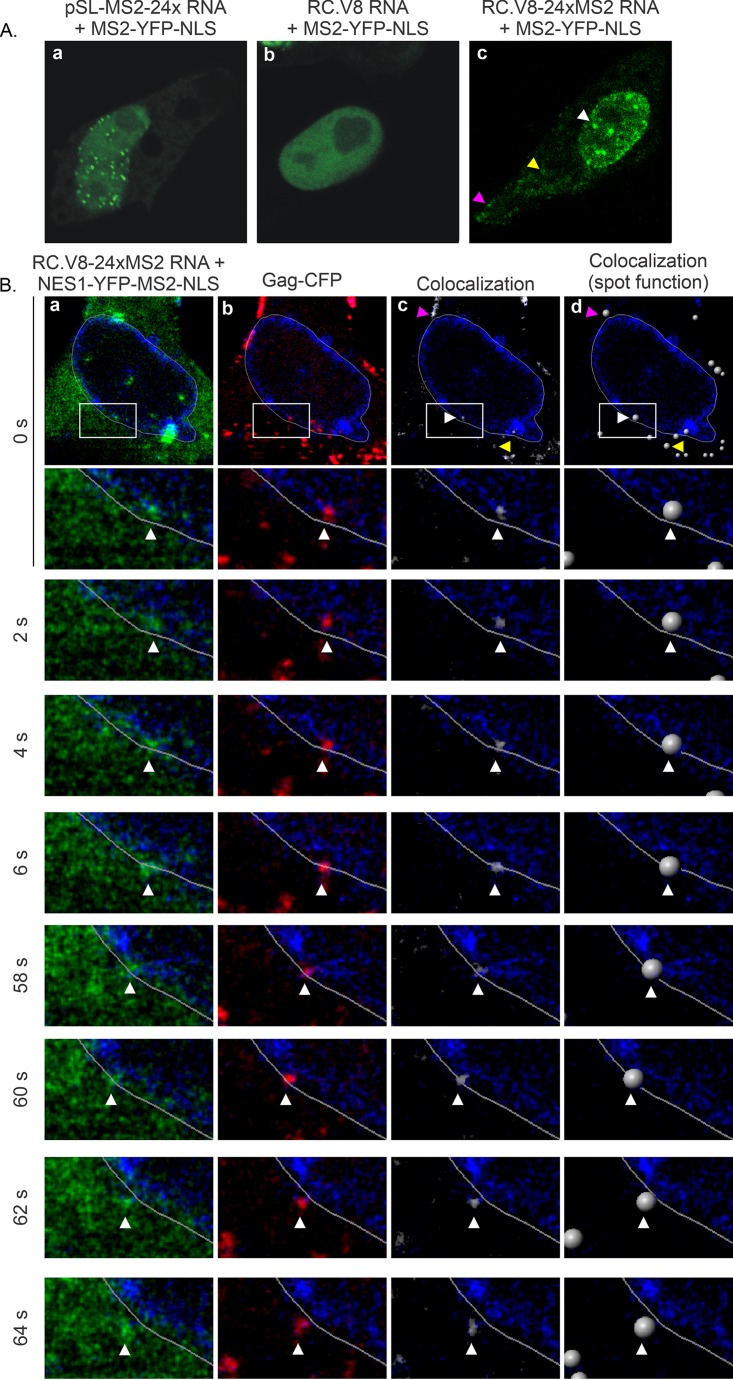
Subcellular localization of MS2-YFP-NLS and wild-type Gag-CFP in QT6 cells. (A) In the presence of the pSL-MS2-24x, MS2-YFP-NLS forms puncta in the nucleus. In the absence of MS2 RNA stem-loops, MS2-YFP-NLS (RC.V8 panel) remains diffuse in the nucleus. When MS2-YFP-NLS is coexpressed with RC.V8-24xMS2, MS2-YFP-NLS foci are present in the nucleus (white arrow), in the cytosol (yellow arrow), and at the plasma membrane (magenta arrow). (B) Various time points from a live-cell time-lapse of QT6 cells were transfected with pRC.V8-24xMS2, pNES1-YFP-MS2-NLS, wild-type pGag-CFP, and pSun1-mCherry. Cells were imaged every 2 s over a period of approximately 10.5 min. vRNA (green) colocalizes (white) with Gag-CFP (red) at the plasma membrane (magenta arrow). (a to c) A focus of NES1-YFP-MS2-NLS-tagged vRNA (arrowhead) (a) and a focus of Gag-CFP (arrowhead) (b) colocalize (c) in the nucleus, which is outlined with Sun1-mCherry (false-colored blue/white outline in all images). (c) Regions of colocalized signal were visualized via the generation of a colocalization channel (white). Colocalized Gag-vRNA foci are indicated by white arrows. (d) The colocalization channel was used to generate spots of Gag-vRNA complexes in the nucleus and the surrounding area that can be tracked over time.

To examine the appearance of the MS2 coat protein in the presence of 24 copies of the MS2 stem-loops in QT6 cells, we cotransfected pSL-MS2-24x (which expresses only the MS2 RNA stem-loop cassette) with pMS2-YFP-NLS. Numerous nuclear RNA foci were visualized, indicating that MS2-containing RNA molecules were labeled cotranscriptionally, as described previously (Fig. 2Aa) (36). As a negative control, we coexpressed MS2-YFP-NLS with RC.V8, which lacks MS2 stem-loops. As expected, MS2-YFP-NLS remained diffuse in the nucleus (Fig. 2Ab). In contrast, when RC.V8-24xMS2 was coexpressed with MS2-YFP-NLS, vRNA was fluorescently labeled upon binding to MS2-YFP-NLS, and numerous foci representing vRNPs were detected in the nucleus (white arrow, Fig. 2Ac), cytosol (yellow arrow), and along the plasma membrane (magenta arrow). This result indicates that the RSV RNA was transported out of the nucleus to sites of virus assembly at the plasma membrane, likely due to the activity of the NES in the Gag p10 domain (compare Fig. 2Aa and c). Together, these data further substantiate the previous conclusion from data presented in Fig. 2B and C that MS2-YFP-NLS binding to the MS2 aptamers in RC.V8-24xMS2 RNA did not interfere with normal vRNA trafficking.

### Colocalization of RSV Gag-CFP with MS2-YFP-NLS-labeled vRNA.

To simultaneously image the distribution of wild-type RSV Gag and vRNA in living cells, QT6 cells expressing RC.V8-24xMS2 RNA with NES1-YFP-MS2-NLS (green; the NES reduces background nuclear fluorescence; Fig. 2Ba) (49), Gag-cyan fluorescent protein (Gag-CFP) (red, Fig. 2Bb), and Sun1-mCherry (blue), as a marker of the inner leaflet of the nuclear envelope (50, 51), were imaged. Gag-CFP (Fig. 2Bb) forms discrete puncta along the plasma membrane, in the cytoplasm, and in the nucleus (1, 2, 12, 31), mimicking the distribution of Gag during infection (1, 2, 11–13, 15, 30, 31). The Imaris colocalization package was used to detect overlapping Gag-vRNA signals (Fig. 2Bc; see also Movie S3, 0 s to 6 s, and Movie S4, 58 s to 64 s, in the supplemental material), and the colocalized foci were transformed into spots using the Imaris spot function (Fig. 2Bd, white balls). Confocal time-lapse images were obtained every 2 s over ∼10.5 min. Single frames are shown at the indicated time points of 2, 4, and 6 s (Movies S1 and S3) and 58, 60, 62, and 64 s (Fig. 2B and Movies S2 and S4). A single confocal slice was imaged to ensure that any vRNP complexes observed would be located within the nuclear plane and would be detected in 2-dimensional space using the Imaris colocalization algorithm. Gag or vRNA complexes that moved out of the nuclear plane would be not be imaged, simplifying the analysis.

10.1128/mBio.00524-20.1 MOVIE S1 Four time points (0 s to 6 s) of a live-cell time-lapse movie of wild-type Gag-CFP and RC.V8-24xMS2 RNA in the nucleus. Live QT6 cells expressing Gag-CFP (red), RC.V8-24xMS2 (green), NES1-YFP-MS2-NLS, and Sun1-mCherry (blue/gray outline) were imaged every 2 s for approximately 10.5 minutes. Eight seconds of the time-lapse is presented in this video. The region of interest is circled. Download Movie S1, MOV file, 0.7 MB. Copyright © 2020 Maldonado et al. 2020 Maldonado et al. This content is distributed under the terms of the Creative Commons Attribution 4.0 International license.

10.1128/mBio.00524-20.2 MOVIE S2 Four time points (58 s to 64 s) of a live-cell time-lapse movie of wild-type Gag-CFP and RC.V8-24xMS2 RNA in the nucleus. Live QT6 cells expressing Gag-CFP (red), RC.V8-24xMS2 (green), NES1-YFP-MS2-NLS, and Sun1-mCherry (blue/gray outline) were imaged every 2 s for approximately 10.5 minutes. Eight seconds of the time-lapse is presented in this video. The region of interest is circled. Download Movie S2, MOV file, 0.9 MB. Copyright © 2020 Maldonado et al. 2020 Maldonado et al. This content is distributed under the terms of the Creative Commons Attribution 4.0 International license.

10.1128/mBio.00524-20.3 MOVIE S3 Four time points (0 s to 6 s) of a live-cell time-lapse movie showing colocalized wild-type Gag-CFP and RC.V8-24xMS2 RNA in the nucleus. Live QT6 cells expressing Gag-CFP, RC.V8-24xMS2, NES1-YFP-MS2-NLS, and Sun1-mCherry (blue/gray outline) were imaged every 2 s for approximately 10.5 minutes. Spots were generated from a colocalization channel (white spheres) created in Imaris (Bitplane) from the same region of time-lapse presented in Movie S1. Eight seconds of the time-lapse is presented in this video. The region of interest is circled. Download Movie S3, MOV file, 0.2 MB. Copyright © 2020 Maldonado et al. 2020 Maldonado et al. This content is distributed under the terms of the Creative Commons Attribution 4.0 International license.

10.1128/mBio.00524-20.4 MOVIE S4 Four time points (58 s to 64 s) of a live-cell time-lapse movie showing colocalized wild-type Gag-CFP and RC.V8-24xMS2 RNA in the nucleus. Live QT6 cells expressing Gag-CFP, RC.V8-24xMS2, NES1-YFP-MS2-NLS, and Sun1-mCherry (blue/gray outline) were imaged every 2 s for approximately 10.5 minutes. Spots were generated from a colocalization channel (white spheres) created in Imaris (Bitplane) from the same region of time-lapse presented in Movie S2. Eight seconds of the time-lapse is presented in this video. The region of interest is circled with a white line. Download Movie S4, MOV file, 0.3 MB. Copyright © 2020 Maldonado et al. 2020 Maldonado et al. This content is distributed under the terms of the Creative Commons Attribution 4.0 International license.

In the top row of images (Fig. 2B, 0 s), RC.V8-24xMS2 RNA and Gag-CFP were colocalized at the plasma membrane (colocalization channels, magenta arrow), in the cytoplasm (yellow arrow), and inside the nucleus (white arrow). In the enlarged image at 0 s, a complex containing RSV RNA overlapped wild-type RSV Gag at approximately 7 o’clock (white arrow) inside the Sun1 signal marking the inner nuclear envelope (Movies S1 and S3). Over time, the vRNP focus initially stayed inside the nuclear envelope (2 s) and then moved through the Sun1 signal during later time points (4 to 60 s) and into the cytoplasm in images taken at 62 to 64 s (Movies S2 and S4). This live-cell confocal imaging time-lapse series demonstrated that wild-type RSV Gag colocalized with RSV vRNA in the nucleus and formed a vRNP complex that trafficked across the nuclear envelope to enter the cytoplasm (Movies S1 to S4).

**FIG 3:**
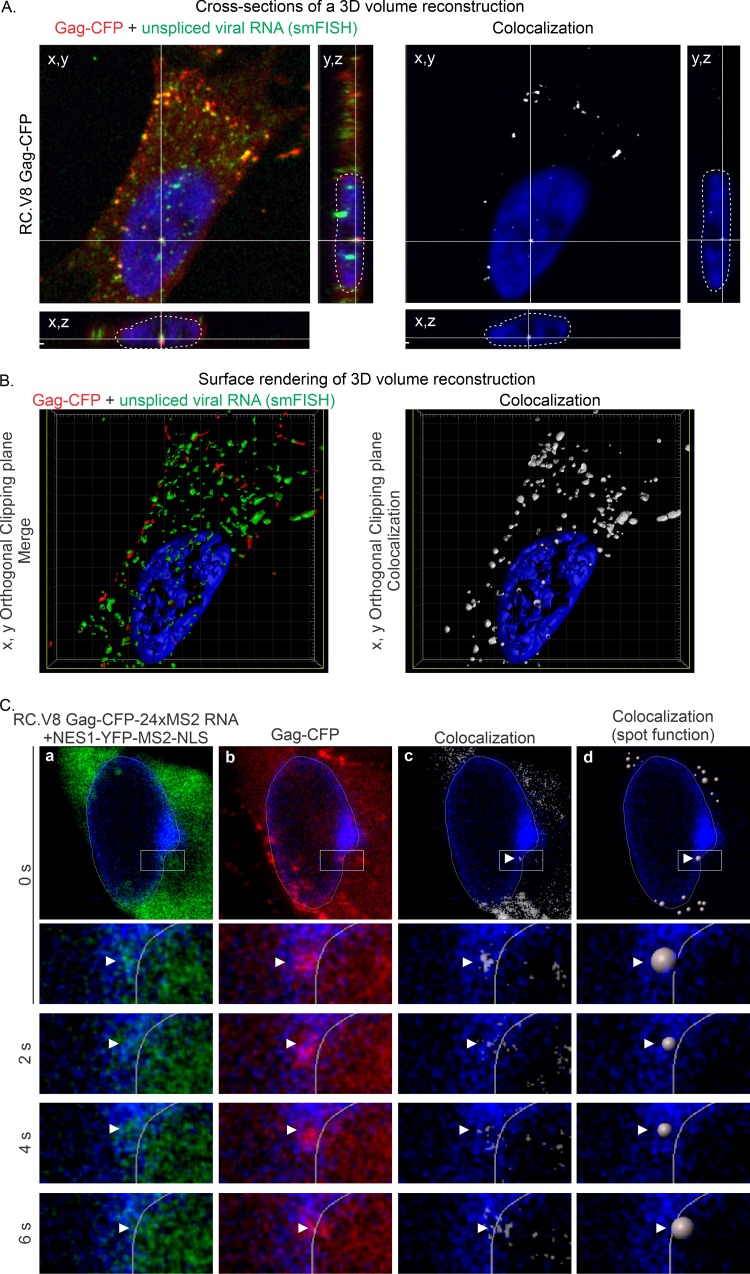
Subcellular localization of wild-type Gag-CFP and unspliced vRNA in QT6 cells. (A) Cross-sections of a z-stack of QT6 cell expressing the proviral pRC.V8 Gag-CFP construct. The Gag-CFP (red) colocalized with unspliced vRNA (green) labeled using RNA smFISH. The crosshairs show a complex of Gag and vRNA that is located in the nucleus. Left, cross-section of an overlay of Gag (red) and vRNA (green). Right, white signal indicates areas of Gag-vRNA colocalization. (B) The z-stack from panel A was generated into a 3D volume surface rendering. Left, *x,y* orthogonal clipping plane of a cell displaying unspliced RSV RNA (green) labeled via smFISH and Gag-CFP (red) complexes within the DAPI-stained nucleus (blue). Right, colocalization between unspliced vRNA and Gag-CFP is displayed as a white surface rendering an *x,y* cut of the same cell. (C) Stills from a live-cell time-lapse movie of QT6 cells transfected with pRC.V8 Gag-CFP-24xMS2, untagged wild-type pGag.ΔPR, pNES1-YFP-MS2-NLS, and pSun1-mCherry. Cells were imaged every 2 s. Unspliced vRNA (green) colocalizes (white) with Gag-CFP (red) in the inner nuclear rim (blue/white outline). (a to c) Foci of NES1-YFP-MS2-NLS-tagged vRNA (arrowhead) (a) and foci of Gag-CFP (arrowhead) (b) colocalize (c) in the nucleus, which is outlined with Sun1-mCherry (false-colored blue in all images). (c) Regions of colocalized signal were visualized via the generation of a colocalization channel (white). Colocalized Gag-vRNA foci are indicated by white arrows. (d) The colocalization channel was used to generate spots of Gag-vRNA complexes in the nucleus and the surrounding area that can be tracked over time.

In the experiments presented in Fig. 2B, pRC.V8-24xMS2 was used to image the trafficking of an infectious proviral construct (Fig. 1C). However, because the MS2 RNA loops were inserted in the exon 3′ of the *env* sequence (Fig. 1A and 2), both spliced and unspliced RSV RNA were labeled. Therefore, to detect only unspliced vRNA complexes, single-molecule fluorescence *in situ* hybridization (smFISH) probes complementary to the *pol* coding region specifically labeled unspliced vRNA produced from a provirus-based construct containing Gag-CFP (pRC.V8 Gag-CFP; Fig. 1A). QT6 cells were transfected, fixed, and hybridized with *pol* smFISH probes to label unspliced vRNA, and a confocal z-series through the entire cell was obtained (Fig. 3A). Reconstruction of the images demonstrated colocalization (yellow) of unspliced RSV RNA (green) and Gag-CFP (red) in the nucleus in three dimensions (3D), as shown by transecting a single vRNP in the *x,y*, *x,z*, and *y,z* planes (Fig. 3A, left). A colocalization channel was also generated, showing the overlap between the Gag and unspliced vRNA signals in the nucleus as a white focus apparent in all 3 planes (Fig. 3A, right). A 3D surface rendering of the z-stack was constructed and an orthogonal clipping plane applied to transect through the center in the *x,y* plane (Fig. 3B and Movie S5). Gag and unspliced vRNA foci were colocalized in the nucleus (Fig. 3B, left), as seen in the colocalization channel (Fig. 3B, right). Furthermore, the 3D rendering also showed that the vRNP complexes were present in the DAPI-poor perichromatin space.

10.1128/mBio.00524-20.5 MOVIE S5 Surface rendering of Gag-CFP and unspliced vRNA in the nucleus. Confocal z-stacks of QT6 cells expressing RC.V8-Gag-CFP were captured and volume and surface renderings created in Imaris. Gag-CFP (red) colocalized with unspliced vRNA (smFISH-green). The cell rotates, and an orthogonal clipping plane transects the cell in the *x,y* plane to show red and green Gag-RNA complexes in the interchromatin spaces of the DAPI staining (blue). A surface rendering was also created of the colocalization channel (white) to better visualize Gag-unspliced RNA complexes in the interchromatin spaces. Download Movie S5, MOV file, 6.4 MB. Copyright © 2020 Maldonado et al. 2020 Maldonado et al. This content is distributed under the terms of the Creative Commons Attribution 4.0 International license.

To specifically visualize the trafficking of unspliced vRNA and Gag-CFP in living cells, we created a proviral construct in which 24 copies of the MS2 cassette were removed from the 3′ end of the genome and repositioned into the *pol* gene, which lies in the intronic region of the unspliced vRNA, downstream of *gag-cfp* (pRC.V8 Gag-CFP-24xMS2; see Fig. 1A). Complexes containing unspliced vRNA labeled with NES1-YFP-MS2-NLS (green) and Gag-CFP (red) were imaged using confocal time-lapse microscopy (Fig. 3C and Movie S6). Untagged wild-type Gag (pGag.ΔPR) was coexpressed to facilitate plasma membrane trafficking of the Gag-CFP fusion protein expressed by the proviral construct pRC.V8 Gag-CFP-24xMS2 (52). At time 0 s (Fig. 3C), unspliced vRNA (panel a) colocalized with Gag (panel b) to form a vRNP (panel c, white-colocalized focus; panel d, white colocalized spot; Movie S7) within the inner nuclear rim outlined by Sun1-mCherry (blue). The colocalized vRNP complex was initially seen inside the nuclear rim (0 s), moving farther toward the cytoplasm at 2 s and 4 s, and emerging outside the Sun1 signal at 6 s (Movie S7). Taken together, these live-cell imaging experiments demonstrated that wild-type RSV Gag colocalized with unspliced vRNA in the nucleus to form a vRNP complex that traversed the nuclear envelope, raising the intriguing possibility that Gag binds to newly synthesized unspliced vRNA in the nucleus as a mechanism to encapsidate the vRNA genome. Additional live-cell imaging experiments will be needed to comprehensively measure the mobility of RSV Gag-vRNA complexes, the directionality and trajectory of complexes, and the dwell time of vRNPs at each stage of nuclear transport.

10.1128/mBio.00524-20.6 MOVIE S6 Four time points (0 s to 6 s) of a live-cell time-lapse movie showing colocalized wild-type Gag-CFP and unspliced RC.V8-Gag-CFP-24xMS2 RNA in the nucleus. Live QT6 cells expressing Gag-CFP in the context of RC.V8 Gag-CFP-24xMS2 (red), NES1-YFP-MS2-NLS (green), untagged wild-type Gag.ΔPR, and Sun1-mCherry (blue/gray outline) were imaged every 2 s for approximately 25 minutes. The region of interest is circled. Download Movie S6, MOV file, 1.0 MB. Copyright © 2020 Maldonado et al. 2020 Maldonado et al. This content is distributed under the terms of the Creative Commons Attribution 4.0 International license.

10.1128/mBio.00524-20.7 MOVIE S7 Four time points (0 s to 6 s) of a live-cell time-lapse movie of wild-type Gag-CFP and unspliced RC.V8-24xMS2 RNA in the nucleus. Live QT6 cells expressing Gag-CFP in the context of RC.V8 Gag-CFP-24xMS2, NES1-YFP-MS2-NLS, untagged wild-type Gag.ΔPR, and Sun1-mCherry (blue/gray outline) were imaged every 2 s for approximately 25 minutes. Spots were generated from a colocalization channel (white spheres) created in Imaris (Bitplane) from the same region of time-lapse presented in Movie S6. Eight seconds of the time-lapse is presented in this video. The region of interest is circled. Download Movie S7, MOV file, 0.4 MB. Copyright © 2020 Maldonado et al. 2020 Maldonado et al. This content is distributed under the terms of the Creative Commons Attribution 4.0 International license.

### RSV Gag colocalizes with unspliced vRNA in infected cells.

**FIG 4:**
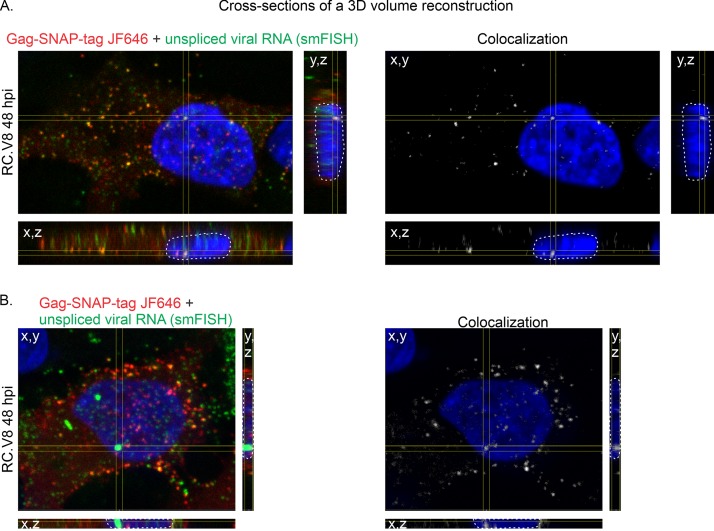
Colocalization between Gag and unspliced vRNA in infected cells. Cross-sections of RC.V8-infected cells transfected with Gag-SNAP-tag JF646 (red) and unspliced vRNA labeled via smFISH (green). (A) An example of a cell containing Gag-unspliced viral RNA complexes in the nucleus, cytoplasm, and plasma membrane. Left, a complex of Gag (red) and unspliced vRNA (green) within the nucleus (white outline) is outlined in the yellow crosshairs. Right, a white colocalization channel was generated between Gag and unspliced vRNA. Foci of colocalization are also present in the cytoplasm and at the plasma membrane. (B) Left, a second example of a cell in which Gag (red) colocalizes with a large unspliced vRNA focus (green) in the nucleus (white outline). Right, a colocalization channel in white shows where the complex resides in the nucleus. Fifteen cells were imaged from three biological replicates.

To address the possibility that the production of Gag and vRNA by transfection of proviral plasmid constructs could lead to differences in the formation of vRNPs compared to that with authentic virus infection, in which vRNA is produced from the proviral integration site, we examined whether Gag colocalized with unspliced vRNA in acutely infected cells. To this end, QT6 cells were incubated with RC.V8 virions for 4 h, transfected with a Gag-SNAP tag (Fig. 1), and incubated with Janelia Fluor 646 (JF646) (53) to detect single Gag molecules. Unspliced vRNA was labeled by using smFISH. Confocal z-stacks were obtained at 48 hpi, and Gag (red) was observed to colocalize with unspliced vRNA (green) in the nucleus, in the cytoplasm, and at the plasma membrane throughout the z-stack in 3 dimensions (Fig. 4A). Colocalization analysis was performed using the Imaris colocalization algorithm, with colocalized vRNP foci shown in white (Fig. 4A and B, right, colocalization channel). In all cases (15 cells imaged), acutely infected cells contained at least one large nuclear RNA focus (mean diameter, 0.636 ± 0.021 μm [SEM]), which may represent a burst of vRNA synthesis at the proviral integration site (54). We observed Gag colocalization with unspliced vRNA at these large RNA foci in 73% of cells (Fig. 4B), as well as at smaller vRNA nuclear foci (colocalization channel, white foci, Fig. 4A and B) distributed throughout the nucleus. A 3D surface rendering of the cell in Fig. 4A was generated, and an orthogonal clipping was applied to the *x,y* plane, demonstrating that the unspliced vRNPs were present in the DAPI-poor perichromatin space, where active transcription takes place (55–58). The localization of unspliced vRNPs to perichromatin in infected cells was similar to that observed in transfected cells (compare Movies S5 and S8). Together, these data indicate that Gag colocalized with unspliced vRNA in the nuclei of acutely infected cells, suggesting that formation of the vRNP complex may occur at the site of ongoing vRNA transcription at the proviral integration site.

10.1128/mBio.00524-20.8 MOVIE S8 Surface rendering of Gag-SNAP tag JF646 and with unspliced vRNA in the nucleus of an acutely infected cell. Confocal z-stacks of a QT6 cell infected with RC.V8 for 48 h and transfected with Gag-SNAP tag JF646 was captured, and volume and surface renderings were created in Imaris. Gag-SNAP tag JF646 (red) colocalized with unspliced vRNA (smFISH-green). An orthogonal clipping plane transects the cell in the *x,y* plane and shows red and green Gag-RNA complexes in the interchromatin spaces of the DAPI staining (blue). A surface rendering was also created of the colocalization channel (white) to better visualize Gag-unspliced RNA complexes in the interchromatin spaces. Download Movie S8, MOV file, 5.4 MB. Copyright © 2020 Maldonado et al. 2020 Maldonado et al. This content is distributed under the terms of the Creative Commons Attribution 4.0 International license.

### RSV Gag directly interacts with unspliced vRNA in discrete nuclear foci.

**FIG 5:**
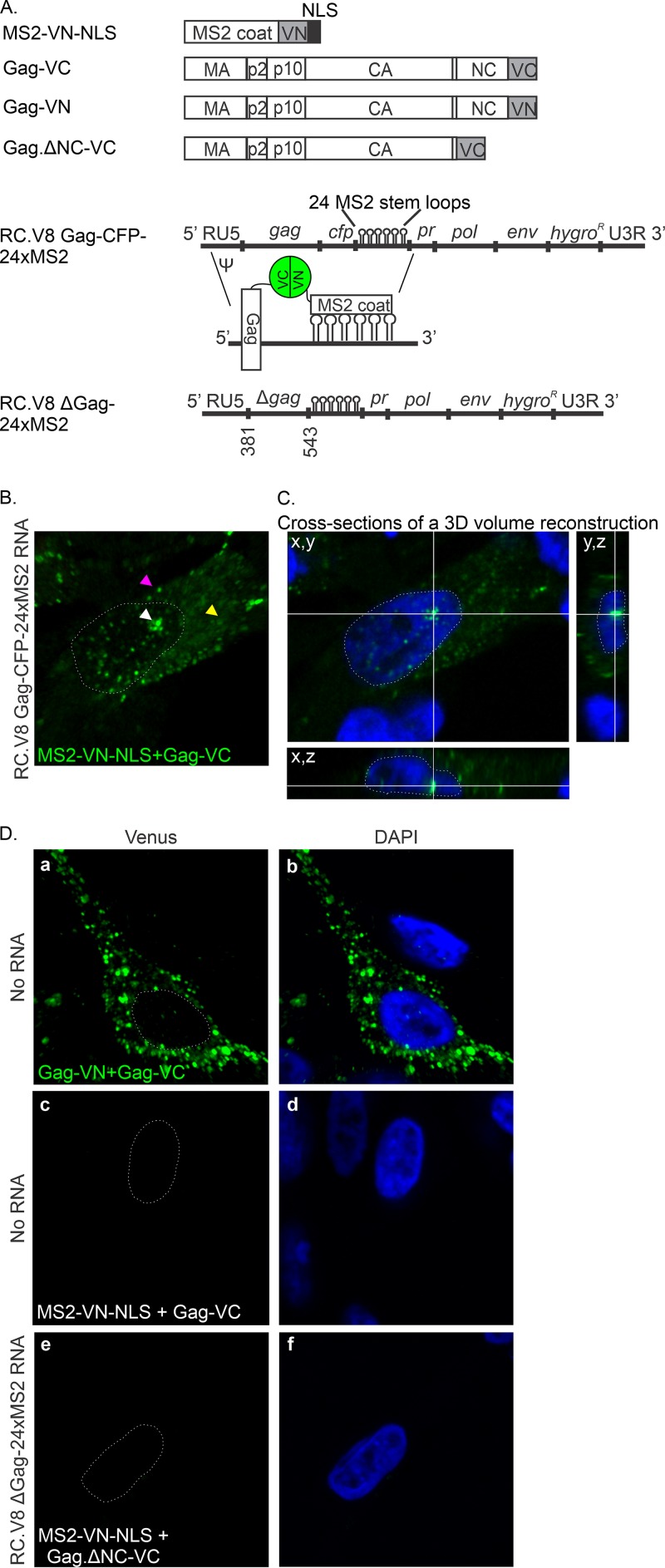
BiFC between Gag and unspliced vRNA. (A) RC.V8 Gag-CFP-24xMS2 contains 24 copies of the MS2 stem-loops between *gag-cfp* and *pr*, allowing for the labeling of only the unspliced vRNA. Because the stem-loops are closer to Ψ, this allows MS2-VN-NLS to bind to Gag-VC if Gag binds to the vRNA, causing a positive BiFC signal. (B) A single optical slice of a QT6 cell expressing RC.V8 Gag-CFP-24xMS2, MS2-VN, and Gag-VC. BiFC foci representing Gag-unspliced vRNA complexes are present in the nucleus (white arrow), in the cytoplasm (yellow arrow), and at the plasma membrane (magenta arrow). (C) A cross-section of a z-stack of the same cell. The crosshairs show a complex of Gag and unspliced vRNA in the nucleus. (D) (a and b) Gag-VN and Gag-VC form BiFC foci (green) in the nucleus, in the cytoplasm, and at the plasma membrane. (c and d) In the absence of RC.V8, Gag-CFP-24xMS2, MS2-VN-NLS, and Gag-VC do not fluoresce. (e and f) BiFC fluorescence was negative when a functional Gag was absent. Combinations expected to have a positive BiFC signal are labeled in green text, and those with negative signal are have white labels.

Although the movement of colocalized Gag-vRNA complexes is highly suggestive of a direct binding event, to examine whether there was direct binding between Gag and unspliced vRNA, we employed two biophysical imaging techniques, bimolecular fluorescence complementation (BiFC) and Förster resonance energy transfer (FRET). For BiFC, the N-terminal half of the Venus fluorophore was fused to the MS2 protein (MS2-VN-NLS), and the C-terminal portion was fused to Gag (Gag-VC; Fig. 5A) (59, 60). When coexpressed, if the N- and C-terminal halves of Venus are in close proximity, they will irreversibly interact and reconstitute the fluorophore. To determine whether unspliced RSV RNA would bridge the binding of MS2-VN with Gag-VC, we coexpressed MS2-VN-NLS, Gag-VC, and RC.V8 Gag-CFP-24xMS2 (Fig. 5A). Confocal images obtained through the nuclear plane revealed the detection of a Venus BiFC signal in the nucleus, in the cytoplasm, and at the plasma membrane (Fig. 5B), indicating that there was very close approximation of Gag and unspliced vRNA. The presence of the Gag-vRNA signal within the nucleus was further examined by generation of a 3D cross-section reconstructed from a confocal z-stack, demonstrating that the BiFC signal was within the nucleus (Fig. 5B, right).

To ensure that the Gag-vRNA BiFC signal was specific, a series of control experiments were performed. Because Gag forms intermolecular protein-protein complexes in the cell (12), we coexpressed Gag-VN and Gag-VC and observed foci in the nucleus, in the cytoplasm, and at the plasma membrane, as expected (Fig. 5Da and b). In the absence of MS2-containing vRNA, MS2-VN-NLS and Gag-VC produced negligible levels of fluorescence (Fig. 5Dc and d). Furthermore, coexpression of a proviral construct that contains the psi packaging signal (Ψ), the MS2 RNA stem-loops (pRC.V8 ΔGag-24xMS2), and NC deletion of Gag (Gag.ΔNC-VC) failed to produce BiFC fluorescence with MS2-VN-NLS, suggesting that NC is required for a positive BiFC signal (Fig. 5De and f). Similarly, coexpression of MS2-VN-NLS and Gag-VC produced negligible fluorescence when expressed with (i) an RSV proviral construct that does not contain MS2 stem-loops; (ii) nonviral RNA containing MS2 loops, or (iii) RSV proviral RNA with noncompatible RNA stem-loops (data not shown). Thus, a BiFC signal was produced only when MS2-VN-NLS and Gag-VC were bound to the same unspliced vRNA molecule in close proximity.

As an alternative to BiFC, we performed FRET acceptor photobleaching (FRET-AB) to examine whether direct binding occurred between Gag-CFP and unspliced MS2-vRNA labeled by MS2-YFP. FRET can occur between two fluorophores (FRET pairs) if the emission spectrum of one fluorophore (“donor”) overlaps the excitation spectrum of another fluorophore (“the acceptor”), such as the FRET pair CFP-YFP, and the fluorophores are in close proximity (61). During FRET-AB, the acceptor fluorophore is bleached, preventing energy transfer from the donor, resulting in an increase in the donor signal. FRET efficiency is calculated by comparing the difference in the donor intensity measures before and after bleaching (see Materials and Methods for details). Because FRET only occurs when the fluorophores are within 10 nm (62), positive FRET efficiency is indicative of direct binding.

**FIG 6:**
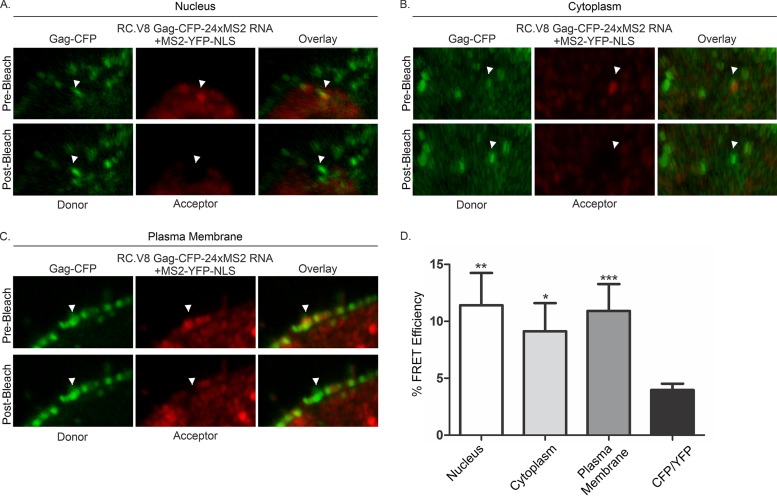
FRET efficiency. All images presented demonstrated the highest FRET efficiency measured during the experiment to better visualize the change in Gag-CFP signal from prebleach to postbleach. (A to C) Examples of FRET between a Gag-CFP (donor) focus and unspliced vRNA labeled with MS2-YFP (acceptor) focus (white arrows) in the nucleus (A), in the cytoplasm (B), and at the plasma membrane (C). (D) The FRET efficiencies of Gag and unspliced vRNA in the nucleus (11% ± 3%; *P* = 0.0031), in the cytoplasm (9% ± 2%, *P* = 0.0180), and at the plasma membrane (11% ± 2%, *P* = 0.0008) were statistically significant over that of the CFP/YFP control (4% ± 0.5%).

In our studies, Gag-CFP (expressed from the pRC.V8 Gag-CFP-24xMS2 proviral plasmid) served as the donor fluorophore, and MS2-YFP-NLS (bound to the unspliced vRNA) was the acceptor. Colocalized foci containing Gag-CFP and unspliced vRNA bound to MS2-YFP that were present in the nucleus, in the cytoplasm, and at the plasma membrane were selected for photobleaching (Fig. 6A to C, respectively). The MS2-YFP-NLS signal in each focus was bleached to ∼10% of the original signal intensity, and the corresponding increase in Gag-CFP intensity was measured using the Leica SP8 FRET-AB wizard. Gag-unspliced vRNA complexes in each subcellular compartment demonstrated positive mean FRET efficiencies that were very similar to one another, with FRET efficiency of 11% ± 3% in the nucleus (*n* = 13 foci), 9% ± 2% in the cytoplasm (*n* = 13 foci), and 11% ± 2% at the plasma membrane (*n* = 11 foci) (Fig. 6D). In each case, the mean FRET efficiency for the unspliced vRNP complexes was significantly higher than in the CFP/YFP controls (4% ± 0.5%, *n* = 21 cells). These results indicate that in each subcellular location, there was evidence for direct binding of Gag to unspliced vRNA within the vRNP complexes.

### Nuclear-restricted RSV Gag specifically associated with unspliced vRNA.

**FIG 7:**
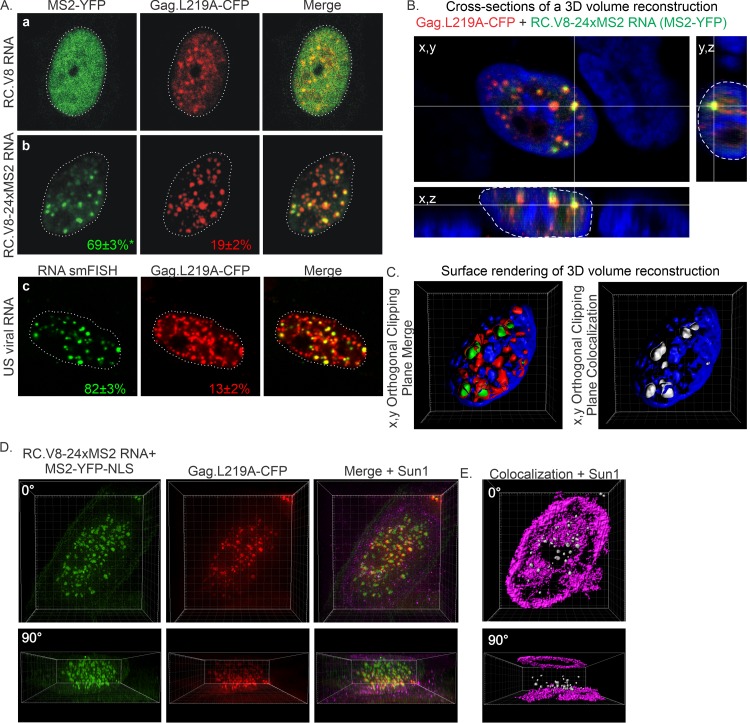
Subcellular localization of vRNA and Gag.L219A in QT6 cells. (A) (a) In the presence of Gag.L219A-CFP, MS2-YFP-NLS remains diffuse in the nucleus in the absence of MS2 stem-loop-containing RNA. (b) RC.V8-24xMS2 RNA foci (9 ± 0.9 foci per nucleus) colocalize with Gag.L219A foci (44 ± 4 foci per nucleus) at a higher level in the nucleus. (c) Unspliced (US) RC.V8-24xMS2 RNAs (11 ± 2 foci per nucleus) colocalized with Gag.L219A-CFP (80 ± 7 foci per nucleus) in the nucleus higher than both spliced and US vRNA (RC.V8-24xMS2). RC.V8-24xMS2 US RNA (FISH) colocalization with Gag.L219A is statistically higher (*P* = 0.0219) than RC.V8-24xMS2 colocalization with Gag.L219A. Statistical analyses were performed in Prism (GraphPad) using an unpaired two-tailed *t* test. (B) A cross-section of a z-stack of a cell expressing RC.V8-24xMS2, MS2-YFP-NLS, and Gag.L219A-CFP and stained with DAPI was generated. The focus of interest (located in between the yellow lines) is outlined in *x,y* (left), *y,z* (right), and *x,z* (bottom) positions. (C) A z-stack of a cell expressing RC.V8-24xMS2, MS2-YFP-NLS, and Gag.L219A-CFP was imaged and used to create a 3D volume surface rendering. Left, *x,y* cut of a cell displaying RC.V8-24xMS2 (green) and Gag.L219A-CFP (red) complexes within the DAPI-stained nucleus (blue). Right, colocalization between the vRNA and Gag.L219A is displayed as a white surface rendering in an *x,y* cut of the same cell. (D) A z-stack the nucleus of a cell expressing RC.V8-24xMS2, MS2-YFP-NLS, Gag.L219A-CFP, and Sun1-mCherry that was imaged using structured illumination microscopy (SIM) was used to create a 3D volume rendering. The 3D rendering is rotated at 90°. (E) A colocalization channel (white) was generated to visualize Gag.L219A-vRNA complexes, and a surface rendering was created from the z-stack of the same cell in panel D.

RSV Gag undergoes active nuclear localization and is exported from the nucleus in a Crm1-dependent manner (1, 11, 14, 31). Although nuclear foci can be observed in cells expressing wild-type RSV Gag (Fig. 2 and 3), they become much larger and more numerous when nuclear export is blocked, either by treatment with the Crm1 inhibitor leptomycin B or when the NES is inactivated by a point mutation (Gag.L219A) (1, 2, 12, 30, 31). We can take advantage of the stability of RSV Gag.L219A nuclear foci (30) to examine the specificity of the Gag-vRNA interaction by quantitatively comparing the relative association of Gag with vRNA versus nonviral RNA. As a negative control, the proviral construct pRC.V8 lacking MS2 binding loops was coexpressed with pGag.L219A-CFP and pMS2-YFP-NLS, and the MS2-YFP-NLS signal (green) remained diffuse in the nucleus due to the lack of MS2 RNA present in these cells (Fig. 7Aa). In contrast, in cells expressing RC.V8-24xMS2 RNA, the MS2-YFP-NLS protein associated with vRNA and formed distinct vRNPs, with quantitative analysis indicating that 69% of vRNA foci colocalized with Gag.L219A puncta (red) (Fig. 7Ab; 50 cells from 8 biological replicates were analyzed). In comparison, 19% of Gag.L219A-CFP foci colocalized with vRNA puncta (Table 1). Because both spliced and unspliced vRNA were labeled with MS2-YFP-NLS in Fig. 7Ab, we performed smFISH to examine only unspliced vRNA associated with Gag.L219A-CFP (Fig. 7Ac). Quantitative two-dimensional colocalization analysis revealed that 82% of unspliced vRNA foci colocalized with Gag.L219A, and 13% of Gag.L219A foci colocalized with unspliced vRNA (Fig. 7A and Table 1; 20 cells from 2 biological replicates). Colocalization of unspliced vRNA (using smFISH) with Gag.L219A was significantly higher than that of MS2-labeled RC.V8-24xMS2 RNA (82% versus 69%, respectively; *P* = 0.0219), most likely due to the increased sensitivity of smFISH compared to that with MS2 labeling of RNA.

**TABLE 1:** Summary of colocalization between vRNA and Gag.L219A foci in the nucleus^*a*^

RNA (label)	Avg ± SEM colocalization (%), *P* value for:	
	RNA with Gag.L219A	Gag.L219A with RNA
RC.V8-24xMS2 US (FISH)	82 ± 3	13 ± 2
RC.V8-24xMS2 US+S (MS2)	69 ± 3, 0.0219	19 ± 2, 0.1853

a Statistical analyses were performed in Prism (GraphPad) using an unpaired two-tailed *t* test.

To determine whether colocalization of Gag-unspliced vRNA foci occurred in the nucleus in 3D, a confocal z-stack was reconstructed (Fig. 7B). Crosshairs drawn through the center of a colocalized focus containing Gag.L219A-CFP and MS2-YFP-NLS-tagged vRNA, which appear yellow in the merged image, demonstrated that the Gag-vRNA signals remained associated in the *x,z* and *y,z* planes, within the confines of the nucleus (white dashed line). A volume rendering of the nuclear z-stack was performed, and an orthogonal clipping plane was applied to the nucleus through the center in the *x,y* plane to show that Gag.L219A-vRNA foci were colocalized in the center of the nucleus (Fig. 7C, left, and Movie S9). These Gag-vRNA complexes were clearly seen in the colocalization channel (Fig. 7C, right, and Movie S9), in which Imaris identified regions of overlap in the red and green channels, displaying them as white. The 3D rendering also showed that the vRNP complexes were nestled within grooves of the DAPI-stained chromatin signal, suggesting that the vRNPs are located in the perichromatin compartment, similar to what was observed with wild-type Gag and unspliced vRNA in transfected and infected cells (Fig. 3B and 4 and Movies S5 and S8). To obtain higher resolution images of the nuclear Gag.L219A-vRNA complexes, we performed structured illumination superresolution microscopy (SIM) (63–67). A series of z-stacks of QT6 cells expressing Gag.L219A-CFP (red), RC.V8-24xMS2 RNA bound by MS2-YFP-NLS (green), and Sun1-mCherry (magenta) were reconstructed to create a 3D volume rendering (Fig. 7D and Movie S10). A colocalization channel for Gag.L219A and vRNA was generated and used to create a surface rending of the colocalized foci present within the nucleus (Fig. 7E). The Gag.L219A-vRNA complexes remained colocalized (white colocalization channel surface, Fig. 7E) in 3D within the nucleus, delineated by Sun1-mCherry (magenta). Thus, visualizing colocalization between Gag.L219A and vRNA further demonstrates interaction. We noticed that the majority of the Gag intranuclear foci were located toward the glass surface of the slide; the reason for this phenomenon is unknown and requires further study.

10.1128/mBio.00524-20.9 MOVIE S9 Surface rendering of Gag.L219A-CFP and RC.V8-24xMS2 vRNA in the nucleus. A z-stack of a cell expressing RC.V8-24xMS2 tagged with MS2-YFP-NLS (green) and Gag.L219A-CFP (red) was imaged and used to create a 3D volume surface rendering. The nucleus rotates and is transected in the *x,y* plane to show Gag-RNA complexes (red and green together) in the interchromatin spaces of the DAPI staining (blue). A surface rendering was also created of the colocalization channel (white) to better visualize Gag-unspliced RNA complexes in the interchromatin spaces. Download Movie S9, MOV file, 5.9 MB. Copyright © 2020 Maldonado et al. 2020 Maldonado et al. This content is distributed under the terms of the Creative Commons Attribution 4.0 International license.

10.1128/mBio.00524-20.10 MOVIE S10 Structured illumination microscopy (SIM) imaging of Gag.L219A-CFP and RC.V8-24xMS2 RNA in the nucleus. SIM imaging was used to image QT6 cells expressing Gag.L219A-CFP (red), RC.V8-24xMS2, MS2-YFP-NLS (green), and Sun1-mCherry (magenta). z-stacks were used to create a 3D volume surface rendering, which was rotated along the *x* axis of the cell. The Gag.L219A (red) and RC.V8-24xMS2 labeled with MS2-YFP-NLS (green) remained associated, with the overlap producing a yellow signal in three dimensions within the outline of the nuclear envelope. Download Movie S10, MOV file, 5.5 MB. Copyright © 2020 Maldonado et al. 2020 Maldonado et al. This content is distributed under the terms of the Creative Commons Attribution 4.0 International license.

## DISCUSSION

In previous studies, we reported that RSV Gag undergoes nuclear shuttling, which is required for efficient packaging of gRNA into virions (1, 2, 10–14, 29–31, 68). Nuclear localization of RSV Gag had been reported much earlier by Enrietto and Erikson (15), who used immunoelectron microscopy to visualize the full-length Gag precursor of Rous-associated virus 2 (RAV-2) in the nucleus in punctate structures and detected the Gag proteins of RAV-2 and RSV strain Schmidt-Ruppin D in nuclear fractions of infected cells. The authors postulated that the nuclear Gag protein could regulate vRNA splicing or could mediate the “selection of newly transcribed vRNA destined to become gRNA in budding virions.”

In the current report, we provide evidence in support of nuclear selection of unspliced vRNA by RSV Gag, suggesting that Gag captures gRNA shortly after its transcription in the nucleus. Using an RSV provirus containing 24 tandem copies of RNA stem-loops from the MS2 bacteriophage (pRC.V8 Gag-CFP-24xMS2; Fig. 1), we directly visualized Gag-unspliced vRNA complexes in the nucleus that trafficked together in a single complex, appearing to cross the nuclear envelope and move outward into the cytoplasm (Fig. 2B and 3C). In addition, we found that Gag colocalized with unspliced viral RNA in acutely infected cells (Fig. 4). These findings provide evidence that Gag may traffic to the proviral integration site to capture unspliced vRNA as genomes at sites of active viral RNA transcription.

We performed several independent experiments which support the conclusion that RSV Gag directly binds unspliced vRNA in the nucleus, in the cytoplasm, and at the plasma membrane. We observed vRNPs to be discrete foci that move together as a single particulate structure across the nuclear envelope outward toward the cytoplasm, suggesting that they have the same kinetics and trajectory and therefore are within a single complex. In the second set of studies, the presence of a BiFC signal arising when Gag and MS2-bound unspliced vRNA came into close proximity is highly suggestive of direct binding. Finally, detecting positive FRET between wild-type Gag and unspliced vRNA within a complex in the nucleus indicates a direct interaction that occurs within 10 nm (Fig. 5 and 6). These findings suggest that RSV Gag directly binds to unspliced vRNA in the nucleus to form a vRNP that remains tightly bound in the cytoplasm and traffics to the plasma membrane. It will be important to examine whether other nucleus-localized retroviral Gag proteins (1, 2, 10–14, 16–31) bind their cognate unspliced vRNAs in the nucleus. Our results provide evidence for nuclear interaction between RSV Gag and unspliced RNA, which represents a single mechanism by which genome packaging could be initiated. It is possible that gRNA recognition and selection for packaging also occur in the cytoplasm, during translation, or at the plasma membrane; these mechanisms are not mutually exclusive. Furthermore, the subcellular location where gRNA selection occurs could differ among retroviruses; therefore, it will be valuable to use a comparative virology approach to studying these differences in the future.

Why would Gag bind unspliced vRNA in the nucleus to initiate the formation of a packaging vRNP complex? The concentration of unspliced vRNA is at its highest level in the nucleus, specifically at or near the transcription site. Therefore, it would be more efficient if Gag were to capture vRNA cotranscriptionally, rather than finding the unspliced gRNA in the cytoplasm where it is mixed with cellular mRNAs. The Ψ sequence is located at the 5′ end of the vRNA and emerges from the transcription site first, allowing Gag to bind shortly after transcription has begun. The mechanism of RSV Gag recruitment to the transcription site is unknown, but this targeting could be mediated by splicing factors themselves, based on previous findings that SF2 and SC35 colocalize with RSV Gag.L219A (30). In addition, there are possible roles for Gag binding to unspliced vRNA in the nucleus that need to be explored, including influencing the ratio of spliced to unspliced RNA, influencing transcription, or promoting chromatin remodeling at the proviral integration site, RNA processing, or nuclear export of nonviral RNAs.

The mechanism by which retroviruses distinguish between unspliced mRNA used for translation and the unspliced gRNA packaged into nascent virions is poorly understood (8). We proposed that RSV Gag may serve a Rev-like function to export the unspliced vRNA from the nucleus for gRNA incorporation into nascent virions. LeBlanc et al. reported that unspliced RSV mRNA is exported from the nucleus through an interaction of the direct repeat (DR) elements with the host Tap/Dbp5 pathway in a Gag-independent manner (69). Because RSV Gag nuclear trafficking is transient, and only a small population of Gag enters the nucleus, it is possible that Gag-Crm1-mediated nuclear export is involved in the export of gRNA and does not play a role in exporting unspliced vRNA used for the translation of viral proteins. We propose that there are two pathways of unspliced RSV RNA export that are temporally separated. Initially, unspliced vRNA may be exported from the nucleus using Tap/Dbp5 (69) to serve as mRNAs for Gag and Gag-Pol translation. After Gag synthesis, Gag may enter the nucleus, bind unspliced vRNA, and export the gRNA for packaging in a Crm1-dependent manner, similarly to HIV-1 Rev, which also functions in conjunction with Crm1 to export unspliced vRNA (70–75).

The data presented herein provide insights into the possible cellular location of the initial Gag-vRNA interaction and provide evidence to support a novel paradigm for retroviral gRNA packaging. Many unanswered questions remain, including whether Gag binds a dimer or monomer of gRNA in the nucleus, how Gag distinguishes unspliced and spliced vRNA, and whether there are additional roles for Gag in the nucleus related to splicing, gene expression, chromatin modification, or nuclear organization? In future studies, we plan to perform 3D tracking to measure the directionality of Gag-vRNP complexes, the kinetics of complex transport between the nucleus and cytoplasm, and the dwell time of vRNPs residing in the nucleoplasm, docked to the inner nuclear leaflet, passing through the nuclear envelope, engaging the outer leaflet of the nuclear membrane, and entering the cytoplasm. This type of comprehensive and quantitative analysis will address many of the important unanswered questions raised by the current study. Further experiments using biophysical, genetic, and imaging techniques will shed light on the answers to these fundamental questions of retroviral biology.

## MATERIALS AND METHODS

### Cells and plasmids.

Experiments were performed using chemically transformed QT6 quail fibroblast cells (obtained from John Wills and Rebecca Craven, Penn State College of Medicine), which were maintained as described previously and transfected using calcium phosphate (32, 33, 48). RSV proviral constructs were derived from pRC.V8 containing the RSV Prague C *gag* gene of pATV8 (33, 76). To create pRC.V8-24xMS2, a BstB1-ClaI restriction fragment from pSL-MS2-24x (from Robert Singer, Albert Einstein College of Medicine) (34, 36) was cloned into the pRC.V8 ClaI site upstream of the hygromycin resistance gene. pMS2-YFP-NLS was obtained from Singer, pNES1-YFP-MS2-NLS containing the Rev NES was obtained from Yaron Shav-Tal, Bar-Ilan University (49), and pSun1-mCherry (pDEST-Sun1-mCherry) was obtained from Jan Karlseder, Salk Institute for Biological Studies (50). Plasmids encoding untagged (Gag.ΔPR) or fluorescently tagged (XFP) wild-type RSV Gag or Gag.L219A were described previously (12, 31, 77). pRC.V8 Gag-CFP was created using Gibson Assembly (78), with fragment 1 digested with PsiI and SalI to remove *ca-env*; fragment 2 encompassing the *ca-nc* region amplified using primers 5′-GTTGATTTTGCCAATCGGCTTATAAAGG-3′ and 5′-CGAGACGGCAGGTGGCTCAGG-3′; fragment 3 containing *cfp* flanked by sequences overlapping the 3′ end of *nc* and the 5′ end of *pr*, generated using primers 5′-CCTGAGCCACCTGCCGTCTCGGCTAGCGGAGGTGGAGGTGTGAGCAAGGGCGAGGAGCT-3′ and 5′-ATGTTCCATTGTCATCGCTAAGCGGCCGCTACGATACTAGTTTCGAATTACTTGTACAGCTCGTCCATGCCGA-3′; and fragment 4 containing *pr-env* amplified using primers 5′-TTAGCGATGACAATGGAACATAAAGATCGCCCCTT-3′ and 5′-AAACTACCTTGTGTGCTGTCGAC-3′. To create pRC.V8 Gag-CFP-24xMS2, MS2 loops were amplified from pCR4-24x MS2SL-stable from Robert Singer (Addgene plasmid no. 31865, http://www.addgene.org/31865/, RRID:Addgene_31865) (36) using primers 5-GCATTTCGAAGAATTTAGCGGCCGCGAATTCGC-3′ and 5′-AGTCATCGATGATTACGCCAAGCTCAGAATTAACC-3′, and inserted into BstBI and SpeI sites in pRC.V8 Gag-CFP. pRC.V8 ΔGag-24xMS2 was created by digesting pRC.V8 Gag-CFP-24xMS2 with SacI and BstBI and inserting a fragment containing the first 160 nucleotides (nt) of MA with the first 2 ATG codons mutated to ATA codons to prevent Gag translation. MS2-VN-NLS was created by amplifying pVenus using primers 5′-ACGCACCGGTCGCCACCATGGTGAGCAAGGGCGAGGAG-3′ and 5′-ACACTGTACATCCTCGATGTTGTGGCGGATCTTGA-3′ and inserting it into AgeI and BsrGI sites in pMS2-YFP-NLS. pGag-VC and pGag.ΔNC-VC were described previously (12).

### Virus infectivity and MS2 packaging assays.

QT6 cells were seeded at 0.5 × 10^6^ in 60-mm dishes and transfected the following day with 5 μg of pRC.V8 or pRC.V8-24xMS2. Cells were collected and passaged every 3 days for 12 days. To assess MS2 coat protein encapsidation, QT6 cells were seeded in 100-mm dishes and transfected with 10 μg of pRC.V8 or pRC.V8-24xMS2 ± 15 μg of pMS2-YFP-NLS. Forty-eight hours later, cells were lysed in RIPA buffer (50 mM Tris-HCl [pH 7.2], 150 mM NaCl, 1% Triton X-100, 0.01% dissolved oxygen concentration [DOC], 0.1% SDS), and the medium was centrifuged and filtered through a 0.22-μm filter. Virus particles were concentrated by ultracentrifugation through a 25% sucrose cushion at 27,000 rpm at 4°C in a SW41 rotor for 1 h and resuspended in TNE buffer (10 mM Tris [pH 7.5], 100 mM NaCl, 1 mM EDTA) or phosphate-buffered saline (PBS).

### Immunoblot analysis.

Viral pellets and equivalent amounts of cell lysates, as determined by a Bradford assay, were separated by SDS-PAGE and transferred to polyvinylidene difluoride (PVDF). Immunoblotting was performed using rabbit anti-RSV polyclonal antibody (79) or mouse ascite fluid against MS2 coat protein (anti-MS2 3H4, a gift from Megerditch Kiledjian, Rutgers University) and detected by chemiluminescence.

### Confocal microscopy.

For fixed-cell experiments, QT6 cells were cultured on 1.5-mm coverslips and transfected with 0.5 μg of pMS2-YFP-NLS (36), 0.5 μg of pGag-CFP, or 1.5 μg of pGag.L219A-CFP/GFP and 3 μg of either pRC.V8, pRC.V8-24xMS2, or pSL-MS2-24x. Cells were fixed 12 to 24 hpt for 15 min with 3.7% paraformaldehyde (PFA) in 2× PHEM buffer [3.6% piperazine-*N,N*′-bis(2-ethanesulfonic acid) (PIPES), 1.3% HEPES, 0.76% EGTA, 0.198% MgSO_4_, pH to 7.0 with 10 M KOH) (80), washed with PBS, DAPI stained, and mounted in antifade reagent (Invitrogen). Slides were imaged on Leica AOBS SP2 or SP8 confocal microscope or DeltaVision Elite deconvolution microscope (GE). Volume and surface renderings and orthogonal clipping planes of z-stacks and histogram adjustments for display purposes were generated using the Imaris image analysis software (Bitplane).

For live-cell time-lapse imaging, cells were seeded on glass-bottom dishes (MatTek Corporation) and transfected with 1 μg of pNES1-YFP-MS2-NLS, either 0.5 μg of pGag-CFP with 3 μg of pRC.V8-24xMS2 (Fig. 2B) or 0.5 μg of pGag.ΔPR with 3 μg of pRC.V8 Gag-CFP-24xMS2 (Fig. 3C), and 0.5 μg of pSun1-mCherry.

Cells were imaged at 16 to 24 hpt and 37°C in clear Dulbecco’s modified Eagle medium (DMEM) with 4.0 mM l-glutamine and 4.5 mg/liter glucose (HyClone) supplemented with 5% fetal bovine serum (FBS), 9% tryptose phosphate broth, and 1% chicken serum on a Leica AOBS SP8 laser scanning confocal at a rate of 1,000 Hz with a pinhole of 2.00 airy units. A frame was taken approximately every 2 s over 10 to 25 min (time was cropped for display). The images were subjected to Gaussian filters and histogram adjustments, and spots of Gag-vRNA complexes in or surrounding the nucleus were generated from a colocalization channel in Imaris.

For BiFC, QT6 cells were transfected with 3 μg of either pRC.V8 Gag-CFP-24xMS2 or pRC.V8 ΔGag-24xMS2, 100 ng of pMS2-VN-NLS, and 100 ng of either pGag-VC or pGag.ΔNC-VC. Blinded data analysis was conducted.

### smFISH labeling of unspliced RSV RNA.

To visualize unspliced vRNA, QT6 cells were seeded and transfected with pRC.V8 Gag-CFP alone or pGag.L219A-GFP with either pRC.V8-24xMS2 or pRC.V8. Cells were fixed at 16 hpt with 3.7% formaldehyde–PBS for 10 min at room temperature and permeabilized in 70% ethanol (EtOH) at 4°C for 1 h. Cells were rehydrated in wash buffer (WB; 10% formamide in 2× [1× SSPE is 0.18 M NaCl, 10 mM NaH_2_PO_4_, and 1 mM EDTA {pH 7.7}] SSPE) for 20 min, and then they were hybridized with 0.5 μl of a 25 μM stock of 46 different Stellaris RNA smFISH probes conjugated to Quasar 570 targeting *pol* (Biosearch) in 100 μl of hybridization buffer (10% dextran sulfate, 2× SSPE, 10% formamide) overnight at 37°C in a humid chamber. Following hybridization, cells were washed for 30 min at 37°C, DAPI stained for 30 min at 37°C, and mounted in ProLong Diamond (Thermo Fisher).

To visualize unspliced vRNA in RC.V8-infected cells, QT6 cells were seeded in a 100-mm dish and transfected with 10 μg RC.V8. The transfection persisted for 1 week, the cells were washed, and medium was collected from the cells. Medium was removed, spun at 2,000 rpm at 25°C for 5 min, and added to QT6 cells that were seeded on coverslips, as described previously. The virions remained on the cells for 4 h before being removed. The infection persisted, and at 32 hpi, cells were transfected with 500 ng of pGag-SNAP tag via the calcium phosphate method. At 47 hpi, cells were labeled with 100 nM SNAP ligand Janelia Fluor 646 (JF646) (53) for 1 h at 37°C. Cells were fixed and subjected to smFISH before being imaged via confocal microscopy. Surface and colocalization channel generation and focus size measurements were conducted in Imaris (Bitplane). Fifteen cells were imaged and analyzed from three biological replicates.

### FRET between Gag and unspliced vRNA.

QT6 cells were seeded and transfected with either 3 μg of pRC.V8 Gag-CFP-24xMS2 and 0.5 μg of pMS2-YFP-NLS or 0.5 μg of pYFP-N1 and 0.5 μg pCFP-N2. FRET acceptor photobleaching was performed with the FRET-AB wizard on the Leica SP8. The acceptor was bleached using 20 to 53% power of the 514-nm laser (argon laser set to 25%) and bleached over 3 to 5 iterations. Only foci with the acceptor (YFP) bleached to approximately 10% of the starting signal and a final donor (CFP) signal intensity (Donor post) greater than or equal to the starting intensity (Donor pre) were analyzed. FRET efficiency was calculated using the following equation:

$$\text{FRET efficiency}=\frac{\left(\text{Donor post}-\text{Donor pre}\right)}{\text{Donor post}}$$

For the sample expressing RC.V8 Gag-CFP-24xMS2 plus MS2-YFP-NLS, colocalized Gag and RNA foci in the nucleus (*n* = 13), in the cytoplasm (*n* = 13), and at the plasma membrane (*n* = 11) were bleached. For the control (YFP + CFP), a similarly sized region of the nucleus (*n* = 21) was bleached. Outliers were identified using the Grubbs’ test (GraphPad), and statistical analysis was performed in Prism (GraphPad) using an unpaired two-tailed *t* test.

### Structured illumination microscopy.

QT6 cells were seeded as described above and transfected with 100 ng pSun1-mCherry, 0.5 μg pMS2-YFP-NLS, 3 μg pRC.V8-24xMS2, and 1.5 μg pGag.L219A-CFP. Twenty-four hours posttransfection, cells were fixed and slides imaged on a DeltaVision OMX microscope (GE). Image manipulation was performed in Imaris, as described above.

### Quantitative colocalization analysis.

Colocalization analysis was performed using custom MatLab (MathWorks) scripts developed by Stephen Lockett, Director of the Optical Microscopy and Image Analysis Laboratory at the National Cancer Institute, Frederick National Lab for Cancer Research (39), and DIPimage (TU Delft). The minimum intensity thresholds for images taken on the Leica SP2 confocal and DeltaVision Elite deconvolution microscopes (GE) were 100 to 150 and 25 to 100 for images obtained on the Leica SP8 confocal with hybrid detectors. A focus was defined as having a minimum radius of 10 pixels. Foci detected in separate channels located within 250 nm (confocal microscope) or 350 nm (deconvolution microscope) were considered colocalized. The average percentage of foci colocalized in each channel was determined. Statistics were performed in Prism using an unpaired two-tailed *t* test.
